# Improvement in quality of life of bitches with malignant mammary neoplasms after unilateral mastectomy and regional lymphadenectomy

**DOI:** 10.29374/2527-2179.bjvm008925

**Published:** 2025-12-16

**Authors:** Mara Tatiani da Silva Bossi, Maria Helena Moreno, Najla Ibrahim Isa Abdel Hadi, Alice Vicenzi, Camila Regina Teixeira de Oliveira, Evandro de Oliveira Rodrigues, Letícia Maria Silva dos Santos, Leonardo Gruchowski, Camila Dalmolin, Fabiana Elias, Fabíola Dalmolin

**Affiliations:** 1 Programa de Pós-Graduação em Saúde, Bem-estar e Produção Animal Sustentável na Fronteira Sul (PPG-SBPAS), Universidade Federal da Fronteira Sul (UFFS), Realeza, SC, Brazil.; 2 Universidade Federal da Fronteira Sul (UFFS), Realeza, SC, Brazil.; 3 Veterinarian autonomous, Arapoti, SC, Brazil.; 4 Univel Centro Universitário, Cascavel, SC, Brazil.; 5 Universidade Federal de Santa Catarina, Florianópolis, SC, Brazil.; 6 Faculdade Mater Dei (UNIMATER), Pato Branco, PR, Brazil.; 7 Universidade Federal da Fronteira Sul (UFFS), Realeza, SC, Brazil.

**Keywords:** animal welfare, cancer pain, HHHHHMM scale, palliative care, pain scale, bem-estar animal, dor associada ao câncer, escala HHHHHMM, cuidados paliativos, escala de dor

## Abstract

Malignant mammary neoplasms (MMNs) are common in bitches, and surgery promotes disease remission and improves quality of life (QoL), which reflects animal welfare. QoL can be assessed using validated scales, answered by the closest caregiver. This study aimed to evaluate the QoL of 15 bitches with MMNs before and after unilateral mastectomy associated to regional lymphadenectomy. The “HHHHHMM Quality of Life Scale” and the "Questionnaire for Evaluating Health-Related Quality of Life in Dogs with Signs of Pain Secondary to Cancer (QEHQ) were administered to the closest caregiver before surgery and 45 days afterward. Differences in QoL before and after surgery were observed according to neoplasm stage, with greater improvement in patients with advanced disease. The HHHHHMM Scale suggested a major improvement in pain-related parameters, while the QEHQ indicated preoperative QoL impairment that was no longer evident after surgery. Unilateral mastectomy and regional lymphadenectomy positively influence the QoL of bitches with MMNs, particularly in advanced stages.

## Resumo

Neoplasmas mamários malignos (NMMs) são comuns em cadelas e a cirurgia promove remissão e/ou melhora a qualidade de vida do paciente, relacionada ao bem-estar animal, acessado por escalas específicas com respostas obtidas do tutor mais próximo. O objetivo deste estudo foi avaliar a qualidade de vida de 15 cadelas com NMMs antes e após a mastectomia unilateral associada à linfadenectomia regional. A "HHHHHMM - Escala de qualidade de vida" e o "Questionário para avaliação da saúde relacionada à qualidade de vida em cães com sinais de dor secundária ao câncer” foram respondidos pelo tutor mais próximo do animal antes e 45 dias após a cirurgia. Diferentes níveis de qualidade de vida foram observados antes e após a cirurgia, e apresentaram correlação com o estadiamento dos neoplasmas; melhores resultados foram observados em animais com estadiamentos avançados. A escala HHHHHMM sugeriu grande diferença relacionada à dor, que diminuiu após a cirurgia. Já o “Questionário para avaliação da saúde relacionada à qualidade de vida em cães com sinais de dor secundária ao câncer” indicou no pré-operatório interferência na qualidade de vida, não observada no pós-operatório. A mastectomia unilateral associada à linfadenectomia regional promove melhora na qualidade de vida de cadelas com NMMs, especialmente aquelas com estadiamento avançado.

## Introduction

As dogs live longer, the incidence of cancer has also increased. Mammary gland neoplasms (MGNs) are the most common type of neoplasm in dogs and represent a major clinical concern (Nosalova et al., 2024). Reported incidence rates vary by study and population. Some registries indicate that MGNs account for 50–70% of all neoplasms in sexually intact dogs (Sorenmo et al., 2020), with approximately 35–50% being malignant (MacPhail & Fossum, 2019). Recent evidence suggests the ratio of malignant to benign MGNs has shifted toward a higher incidence of malignancy, similar to the trend observed in women (Sorenmo et al., 2020).

Affected bitches are typically middle-aged or older and belong to either small or large breeds (MacPhail & Fossum, 2019; Sorenmo et al., 2019). The etiology is multifactorial and includes genetic, environmental, infectious, and nutritional influences. Many of these factors are hormone-dependent and can be prevented through early ovariohysterectomy (Cassali et al., 2020).

Diagnosis and staging are performed using the modified TNM (tumor–node–metastasis) system, which consider tumor size, the presence or absence of inflammatory carcinoma, adherence to the adjacent skin (T), lymph node involvement (N), and the presence or absence of distant metastasis (M) (Cassali et al., 2024; Nosalova et al., 2024). Evaluation involves clinical and surgical examination, particularly in cases of lymphadenopathy, when lymph nodes are palpable, as well as assessment for distant metastases in the lungs, liver, spleen, kidneys, bones, and distant lymph nodes (Cassali et al., 2024).

Treatment options include radiotherapy, hormonal therapy, chemotherapy, targeted therapy, virotherapy (Nosalova et al., 2024), and surgery (Cassali et al., 2024). Although surgery, radiotherapy, and chemotherapy can be effective, they also carry the potential of adverse effects. Moreover, geriatric patients often respond unpredictably to treatment and may experience heightened sensitivity to dietary changes, anesthesia, surgical procedures, and the toxicities associated with chemotherapy and radiotherapy (Sorenmo et al., 2020).

Surgery is the primary treatment, except in cases of inflammatory carcinoma or distant metastasis. The incision margins are determined by the characteristics of the neoplasm, with tumor size and invasiveness playing key roles in achieving cure, improving quality of life (QoL), and altering disease progression. Unilateral mastectomy is indicated if multiple neoplasms are present along the mammary chain, as it reduces the risk of local recurrence and is less time-consuming and traumatic than performing multiple lumpectomies or mastectomies (MacPhail & Fossum, 2019).

QoL assessment in dogs has been increasingly discussed (Smith et al., 2019). Although physical examination provides valuable information, it alone is unlikely to accurately represent the variability in daily pain, QoL, or neoplasm evolution (Williams & MacDonald-Dickinson, 2023). Pain assessment can be supported through structured questionnaires featuring yes/no or multiple-choice responses that evaluate patient's welfare, considering across physical, social, and emotional dimensions.

In oncology, assessing QoL is essential for identifying differences in patient responses to neoplasms, monitoring changes in clinical signs, and evaluating treatment outcomes (Yazbek & Fantoni, 2005). The HHHHHMM Scale facilitates this assessment by evaluating parameters such as hurt, hunger, hydration, hygiene, happiness, mobility, and the balance between good and bad days. It is a user-friendly tool that guides caregivers in evaluating pet QoL (Villalobos, 2011). The scale has been validated in Italian (Testoni et al., 2023) and has also been shown to be useful for Portuguese speakers in assessing the effectiveness of treatments administered to their animals (Mencalha et al., 2019).

The Questionnaire for Evaluating Health-Related Quality of Life in Dogs with Signs of Pain Secondary to Cancer (QEHQ) (Yazbek & Fantoni, 2005) is a validated Portuguese-language scale designed to assess dogs experiencing chronic pain secondary to cancer.

The aim of this study was to evaluate the QoL of bitches with malignant mammary neoplasms (MMNs) before and 45 days after unilateral mastectomy and regional lymphadenectomy, using the HHHHHMM and QEHQ scales.

## Materials and methods

### Animals and inclusion criteria

This study was approved by the Ethics Committee on the Use of Animals (protocol number 23205.002116/2018-10) and the Research Ethics Committee (protocol number 91952518.3.0000.5564). Informed consent was obtained from all animal guardians, who signed forms acknowledging the procedures and associated risks.

Fifteen bitches with cytological confirmation or clinical signs of malignancy were included. Evaluation consisted of anamnesis, physical examination, and laboratory testing, including total blood count, serum creatinine, urea, alkaline phosphatase, alanine aminotransferase, and albumin. MMNs were measured with a caliper (mm), and distant metastases were assessed using thoracic radiography (three views) and abdominal ultrasonography. Based on these data, neoplasm staging was performed according to Cassali et al. (2024). Dogs classified as stages I to IV were included, whereas those with stage V disease were excluded because they required alternative treatment approaches.

To confirm malignancy, the excised lymph nodes and mammary glands were fixed in 10% formalin and examined histopathologically. Histological slides were prepared as described by Caputo et al. (2010), followed by morphological classification and grading according to Zappulli et al. (2019). Lymph nodes were evaluated using hematoxylin and eosin staining (Table 1).

**Table 1:** Weight, age, breed, spay history, contraceptive use, neoplasm size, diagnosis by HE staining, lymph node metastasis (axillary and inguinal), and clinical and surgical TNM staging of bitches with malignant mammary neoplasms.

**N**	**Weight (kg)**	**Age (year)**	**Breed**	**Spayed**	**Contraceptive use**	**Neoplasm dimensions (cm)**	**Metastasis (HE)**	**Diagnosis (HE)**	**Clinical TNM stage**	**Surgical TNM stage**
1	19.6	10	Mongrel	No	Yes	M4 6×4.6×4.2 M5 11×15×8.9	LAx: – LIn: NE	M1, M2, and M3: Epitheliosis M4: Carcinoma tumor mixed degree I M5: Sarcoma*	T3N0M0 IV	T3N1M0 III
2	10.5	10	Mongrel	No	Yes	M5 9.1×9.8×7.5 M4 4×2.5×1	LAx: – LIn: NE	M1: Adenosis M2: Ductal hyperplasia M3: Carcinoma mixed degree I M4: Carcinoma complex degree I M5: Carcinoma tumor mixed degree I	T3N0M0 III	T3N0M0 III
3	15.9	6	Mongrel	No	Yes	M3 4×6.5×3.5	LAx: NE LIn: +	M1, M2, and M4: Ductal ectasia M3: Carcinoma tumor mixed degree I M5: Adenoma	T2N1M0 IV	T2N1M0 IV
4	7.9	8	Dachshund	No	No	M2 0.5×0.5×0.5 M41.5×1.5×1.5 M5 0.5×0.5×0.5	LAx: – LIn: –	M2: Carcinoma tubular degree I M3: Carcinoma tubular degree I; carcinoma complex degree II M4: Carcinoma tumor mixed degree I M5: Carcinoma cystic-papillary degree I	T1N0M0 I	T1N0M0 I
5	22.5	5	Boxer	No	No	M2 2.9×1.9×0.9	LAx: – LIn: NE	M1: Carcinoma complex degree I M2: Carcinoma tumor mixed degree I M3: Duct papillomatosis-associated adenosis M4: Epitheliosis-associated adenosis M5: Carcinoma tubulopapillary; carcinoma solid complex type degree II	T1N0M0 I	T1N0M0 I
6	4.45	8	Mongrel	Yes	No	M2 4.3×3.6×2.2	LAx: NE LIn: –	M1: Duct ectasia M2: Carcinoma micropapillary invasive degree I M3: Complex adenoma M4: Intraductal papillary adenoma	T2N1M0 IV	T2N1M0 IV
7	6.1	12	Poodle	No	Yes	M5 4.1×2.1×1.8	LAx: – LIn: NE	M1: Epitheliosis M3: Complex adenoma M4: Carcinoma tubulopapillary degree I M5: Carcinoma tumor mixed degree I	T2N0M0 II	T2N0M0 II
8	5	8	Poodle	Yes	yes	M5 5×4×2.5	Lax: NE LIn: NE	M2, M3, and M4: Carcinoma tubular degree II M5: Carcinoma tubulopapillary degree I	T2N0M0 II	T2N0M0 II
9	2.75	8	Poodle Toy	Yes	No	M1 1.5×2×0.5 M3 1×1×1 M5 3.9×2.6×1.8	LAx: – LIn: NE	M1: Carcinoma complex degree I M2 and M4: Duct ectasia M3 and M5: Carcinoma mixed degree I	T2N0M0 II	T2N0M0 II
10	21	11	Mongrel	No	Yes	M2 8.2×8.2×8.2	LAx: – LIn: NE	M2: Carcinoma complex degree I M3: Adenosis M4 and M5: Ductal adenoma	T2N0M0 IV	T2N0M0 III
11	3	9	Yorkshire Terrier	Yes	No	M2 2×1.5×0.5	LAx: – Lin: NE	M1 and M4: Epitheliosis M2: Carcinoma complex degree II M3: Adenosis	T1N0M0 I	T1N0M0 I
12	4.6	18	Mongrel	No	Yes	M1 0.1×0.8×0.5 M5 1.8×1.9×1.3	LAx: – LIn: NE	M4: Carcinoma tubulopapillary degree I and carcinoma complex degree I M5: Carcinoma tubulopapillary degree I	T3N1M0 II	T3N0M0 II
13	5.9	11	Lhasa Apso	No	Yes	M4 4.1×4.5×3.2 M5 4×4.1×1.2	LAx: – LIn: –	M1 and M2: No alteration M4: Tumor mixed benign M5: Carcinoma complex degree I	T2N0M0 II	T2N0M0 II
14	8.8	12	Pincher	No	No	M2 1×0.8×0.3 M3 3.8×4.4×3.9 M4-M5 4.9×9.1×8.5	Lax: NE LIn: NE	M2: Carcinoma tubulopapillary degree I M3: Carcinoma complex degree I M4D: Carcinoma tubulocystic papillary degree I M5D: Carcinoma cystic-papillary degree I	T3N0M0 III	T3N0M0 III
15	13.7	11	Poodle	No	Yes	M3-M4 5×5×2.5 M5 1×1×1	Lax: NE LIn: NE	M2: Adenosis M3 and M5: Carcinoma tubulopapillary degree I M4: Adenoma	T2N0M0 III	T2N0M0 III

LAx = axillary lymph node; LIn = inguinal lymph node; – = negative; + = positive; NE = not evaluated; * = to be confirmed; HE = hematoxylin and eosin.

### Data collection

The total score of QoL was used to evaluate the impact of the surgical treatment. Before surgery and 45 days afterward, the same caregivers completed the HHHHHMM Scale (Villalobos, 2011) and the QEHQ (Yazbek & Fantoni, 2005). Both instruments were completed in Portuguese by native speakers and administered by bilingual (Portuguese/English) facilitators. The HHHHHMM Scale was freely translated into Portuguese by two bilingual veterinarians experienced in pain recognition. When necessary, caregivers received clarification of technical terms from the attending veterinarian, without influencing their responses. After the second evaluation, conducted 45 days postoperatively, key QoL aspects were discussed with the owners, and individualized treatment plans were provided when required.

### Anesthesia and surgical procedures

After an eight-hour fast for solids and a two-hour fast for liquids, patients received acepromazine (Acepran 0.2%, Vetnil; 0.02 mg/kg by intramuscular injection), midazolam (Midazolam, Teuto; 0.3 mg/kg by intramuscular injection), and tramadol hydrochloride (Tramadol, Teuto; 5 mg/kg by intramuscular injection). Anesthesia was induced with propofol (Propovan, Cristália; 4 mg/kg administered intravenously) and maintained through tracheal intubation with isoflurane (Isoforine, Cristália) vaporized in 100% oxygen, while blood pressure was kept between 70 and 80 mmHg. Supportive therapy included meloxicam (Maxican 0.2%, Ouro Fino; 0.2 mg/kg administered subcutaneously) and cephalothin (Cefalotina Sódica, Teuto; 30 mg/kg administered intravenously).

After antisepsis and placement of sterile drapes, methylene blue (Azul de metileno 2%, InjectCenter; 0.03 mg/kg) was injected intradermally around the cranial thoracic mammary gland on the same side as the affected mammary chain. The procedure began with excision of the axillary skin, followed by subcutaneous and muscular dissection. The stained lymphatic vessels facilitated localization of the lymph nodes. Lymphatic and blood vessels were ligated using polyglactin 910. The muscle and subcutaneous tissues were closed with interrupted cross sutures using the same material. Skin closure was performed with interrupted mattress sutures using nylon.

A skin incision was made to expose the cranial and caudal epigastric vessels, which were identified, dissected, and doubly ligated with polyglactin 910. A cutaneous incision was then extended around the mammary chain, maintaining a minimum margin of 1 cm, followed by careful dissection of the mammary tissue near the inguinal lymph node. Additional hemostasis was achieved through vessel torsion or ligation with polyglactin 910. The surgical wound was irrigated with warm Ringer’s lactate solution and closed in layers: the subcutaneous tissue was approximated with a simple continuous invaginating suture pattern using polyglactin 910, and the skin was closed with interrupted horizontal mattress sutures using nylon. Suture caliber was adjusted according to the size of each patient. Immediately after surgery, a mixture of lidocaine hydrochloride (Hypocaina, Hypofarma; 5 mg/kg) and bupivacaine hydrochloride (Cloridrato de Bupivacaína, Hypofarma; 4 mg/kg), diluted in 10 mL of 0.9% sodium chloride, was infiltrated along the incision line for local analgesia.

After anesthetic recovery, the patients were discharged, and caregivers received instructions for postoperative care. The prescribed regimen included wound cleaning, bandage protection, topical application of 0.2% chlorhexidine, and the use of surgical clothing twice daily for 10 days. Medication included meloxicam (0.1 mg/kg administered orally [PO] once daily for four days), tramadol hydrochloride (5 mg/kg PO three times daily for five days), dipyrone hydrochloride (25 mg/kg PO three times daily for five days), and cephalexin (30 mg/kg PO twice daily for 10 days). Ten days after surgery, patients were re-evaluated, and skin sutures were removed.

### Statistical analysis

Data distribution was evaluated using the F-test for variance (The Jamovi Project, 2022). For parameters related to clinical staging, means were compared using the Tukey test. Dependent variables were assessed with the Shapiro–Wilk test. A *p*-value of less than 0.05 was considered statistically significant.

## Results

The patients had a mean body weight of 10.39 ± 6.6 kg and a mean age of 9.8 ± 3.08 years. Delayed presentation and the use of contraceptives contributed to the long-term progression of MMNs. One animal had been recently adopted, and information regarding contraceptive use was unavailable for this case. Eight patients (8/15; 53.33%) had received contraceptives at least once, and all of them (8/8; 100%) developed MMNs. Among the 15 dogs, 11 (73.33%) were intact bitches that presented with malignant tumors, while all spayed dogs (4/4; 100%) also showed malignancy. All animals resided within 2 km of agricultural areas.

Complete anesthetic and surgical recovery was achieved in all patients, with no postoperative complications observed. All dogs had at least one malignant neoplasm (15/15; 100%). On palpation, 26 nodes were identified (1.73 per animal), whereas histopathological examination revealed 61 lesions (4.06 per animal). Clinically, 47 mammary glands appeared normal, yet histopathology confirmed the absence of lesions in only nine. The fourth mammary glands were the most frequently affected (15/61; 24.59%). Malignant neoplasms were predominant (34/61; 55.73%), with mixed tumor carcinoma (9/34; 26.47%), complex carcinoma (7/34; 20.58%), and tubulopapillary carcinoma (6/34; 17.64%) being the most common subtypes. Histological grade I tumors represented the majority (28/34; 82.35%). Pre-neoplastic lesions were the second most frequent (19/61; 31.14%), whereas benign lesions were the least common (8/61; 13.11%) and included adenomas, complex adenomas, and ductal adenomas. The identified neoplasms were well-differentiated and showed minimal disruption of cellular architecture.

Ten axillary and four inguinal lymph nodes were excised. Among the axillary group, five dogs (5/15) did not undergo lymph node removal. Of the 10 axillary nodes evaluated, none showed evidence of metastasis. Among the four inguinal lymph nodes examined, one exhibited metastatic involvement. Based on clinical evaluation, four dogs were classified as TNM stage IV, three as stage III, five as stage II, and three as stage I. Among the 12 patients whose lymph nodes were histologically evaluated, staging was modified in two cases. In Animal 1, the axillary lymph node was enlarged on palpation, but histopathology revealed no metastasis, resulting in a change from stage IV to stage III. In Animal 10, the inguinal lymph node was clinically enlarged and initially classified as stage IV; however, histopathology confirmed the absence of metastasis, leading to reclassification as stage III.

The HHHHHMM Scale showed that patients with distinct clinical stages had significantly different scores before surgery (Day 0; *p* < 0.05), with QoL decreasing as clinical stage increased (Table 2). After surgery, no significant differences were observed among patients with different stages (Table 3). Comparison of scores between Day 0 and Day 45 revealed significant improvement (*p* = 0.02) in dogs classified as stages II, III, and IV, with lower scores at Day 0 and higher scores at Day 45. Dogs classified as stage I showed no significant difference between the two evaluations.

**Table 2:** Quality of life scores on the HHHHHMM Scale before surgery (Day 0) and 45 days after surgery (Day 45) in bitches with malignant mammary neoplasms undergoing unilateral mastectomy, according to clinical TNM staging.

**Clinical TNM stage**	**Day 0**	**Day 45**
I	64^a^	66.8^a^
II	56.3^ab^	64.5^a^
III	56^ab^	67^a^
IV	45^b^	66.2^a^
*p*-value	0.0152	0.7894

a,b Different letters in the same column indicate significant differences (*p* < 0.05).

**Table 3:** Average difference in scores at Day 0 and Day 45 on the HHHHHMM Scale and the QEHQ in bitches with malignant mammary neoplasms at different clinical TNM stages following surgery.

**Clinical TNM stage**	**Day 0**	**Day 45**
I	31.5^a^	32.5^a^
II	30.25^a^	30.75^a^
III	26^ab^	32^a^
IV	24^b^	32.6^a^
*p*-value	0.0152	0.7894

a,b Different letters in the same column indicate significant differences (*p* < 0.05).

The initial QEHQ assessment showed differences in QoL among bitches with different clinical stages, which were no longer observed at Day 45 (Table 3). A significant difference in QEHQ scores between Day 0 and Day 45 (*p* = 0.05) was found in dogs classified as stages III and IV, with lower scores before surgery and higher scores afterward.

When comparing the change in scores between Day 0 and Day 45, dogs in stages III and IV showed the greatest improvement after surgery on both the HHHHHMM and QEHQ scales (Table 4).

**Table 4:** Average difference in scores between Day 0 and Day 45 on the HHHHHMM Scale and the QEHQ in bitches with malignant mammary neoplasms undergoing unilateral mastectomy, stratified by clinical TNM stage.

**Clinical TNM**	**HHHHHMM**	**QEHQ**
I	2.8^b^	0.75^bc^
II	8.3^ab^	2.5^c^
III	11^ab^	6^ab^
IV	21.2^a^	8.6^a^
*p*-value	0.0035	0.0007

a,b,c Different letters in the same column indicate significant differences (*p* < 0.05).

On the HHHHHMM scale, the lowest preoperative scores were recorded for pain (7.73 ± 2.56), the ratio of good to bad days (7.06 ± 1.91), and hunger (7.73 ± 2.40). After 45 days, these parameters significantly improved to 9.01 ± 1.82, 9.04 ± 1.25, and 9.56 ± 0.61, respectively (maximum possible score: 10). On the QEHQ, the lowest baseline scores were observed for disease interference with QoL (1.20 ± 1.10), fatigue (1.84 ± 0.87), and pain (1.80 ± 0.54). After 45 days, these values improved to 2.60 ± 0.61, 2.00 ± 0.51, and 2.46 ± 0.61, respectively (maximum possible score: 3).

## Discussion

The weight and age of the animals included in this study were consistent with previous reports, indicating that middle-aged and elderly dogs (8–10 years old) are most commonly affected by MMNs (Nosalova et al., 2024). The development of MMNs is multifactorial and influenced by genetic, environmental, nutritional, and hormonal factors (Burrai et al., 2020; Vazquez et al., 2023). Genetic predisposition (Burrai et al., 2020; Sorenmo et al., 2019), obesity, aging, and exposure to pesticides and other pollutants are recognized contributors. Hormonal imbalances, particularly those involving estrogen, play a major role in the development and progression of MGNs in dogs (Sorenmo et al., 2019), and all of these factors were part of the clinical histories of the patients in this study.

The contraceptives identified in this study have been associated with the induction of cellular dysplasia, hyperplasia, and neoplastic progression (Silva et al., 2019). Regarding reproductive status, MMNs are more frequent in intact bitches, as lifelong hormonal fluctuations increase the likelihood of cellular mutations (Nunes et al., 2017). Although one analysis reported that ovariohysterectomy does not significantly influence the development of MMNs, hormonal variations were still shown to affect tumor behavior (Kristiansen et al., 2016). Early ovariohysterectomy is generally recommended as a protective measure to reduce the incidence of mammary neoplasms (Vazquez et al., 2023). However, the region where this study was conducted has experienced a longstanding shortage of veterinary care, which likely contributed to the observed findings.

All animals in this study lived in proximity to agricultural areas. Western Paraná is known for its extensive use of pesticides (Gaboardi et al., 2019), highlighting the relevance of the One Health concept and its interconnected implications for human and animal health. A relationship between pesticide exposure and MMNs in humans has been reported (Albring et al., 2023), and chronic exposure has been associated with reduced levels of several cytokines, impairing the ability of the immune system to eliminate cancer cells (Santos et al., 2024). Furthermore, other studies have described increased lymphatic invasion and cellular proliferation in exposed individuals, indicating more aggressive neoplasms and poorer prognoses (Santos et al., 2024). Animals likely experience similar effects as humans under comparable environmental conditions.

In women, lymph node mapping has transformed the management of MMNs. Because canine mammary carcinoma is considered a model for the human disease, the staging system used in women should also be applied to dogs (Vazquez et al., 2023), as implemented in this study. Although thoracic computed tomography offers greater sensitivity than radiography for detecting pulmonary nodules, its use is often limited by cost and availability (Sorenmo et al., 2020), which was also the case in this study.

Surgery is the treatment of choice for MMNs (MacPhail & Fossum, 2019; Vazquez et al., 2023), and its objectives should be defined based on disease staging and caregiver decision. In some cases, prophylactic mastectomy of clinically normal glands, in addition to the affected ones, should be considered (Sorenmo et al., 2020). This approach is particularly relevant when pre-neoplastic lesions are present in macroscopically normal glands adjacent to affected ones or when multiple lesions occur within the same chain, as it helps reduce surgical trauma (MacPhail & Fossum, 2019). Both situations were observed in this study.

Regarding the clinically and histopathologically affected glands, the findings align with a previous study reporting that among 314 glands palpated, 138 were affected and 176 appeared normal; however, microscopic examination revealed that only a few had no alterations (Sorenmo et al., 2019). Bitches with MMNs often develop multiple lesions, which increases the likelihood of new tumor formation. Consequently, unilateral mastectomy remains the treatment of choice, as it removes visible lesions, prevents new ones, and addresses those not clinically detectable (Vazquez et al., 2023).

The fourth mammary glands were the most frequently affected in this study, consistent with previous findings (Cassali et al., 2024; Silva et al., 2019). This supports evidence that the abdominal and inguinal glands are most commonly involved, with M4 representing the primary site. This pattern is attributed to the larger amount of glandular parenchyma in this region, which permits greater cellular proliferation (Silva et al., 2019).

The most common malignant lesion identified in this study, mixed tumor carcinoma, was consistent with previous findings (Zappulli et al., 2019), as was the predominance of histological grade I tumors (Zappulli et al., 2019). The observed pre-neoplastic lesions have the potential to progress to neoplasia (Cassali et al., 2024; Vazquez et al., 2023). Adenomas, complex adenomas, and ductal adenomas identified here differ from those reported in another study, which described benign mixed and complex neoplasms (Silva et al., 2019). Regarding the well-differentiated lesions and minimal architectural alterations observed, benign mixed neoplasms can undergo malignant transformation over time, evolving into mixed tumor carcinoma, carcinosarcoma, or mixed tumor sarcoma (Cassali et al., 2024; Gamba et al., 2017). Early surgical excision is therefore essential to prevent this progression.

Axillary lymph node identification was challenging in some patients owing to the absence of methylene blue staining. Inguinal lymph nodes were also frequently undetected, likely because of the presence of large neoplasms involving M4 and M5, which complicates surgical staging in advanced MMNs. Two patients had their clinical stage revised after surgery, emphasizing the importance of postoperative histopathological evaluation for accurate treatment planning (Sorenmo et al., 2019; Vazquez et al., 2023). Despite the low number of metastases observed, immunohistochemistry remains a more effective method for detecting micrometastases and isolated metastatic foci. However, the high cost of this technique limits its accessibility for many caregivers (Sorenmo et al., 2019), in contrast to routine hematoxylin and eosin staining, which was used in this study.

More than half of the patients of this study presented with advanced-stage disease. Tumor size is one of the factors most strongly associated with clinical prognosis and malignancy, with larger neoplasms exhibiting greater aggressiveness than smaller ones (Burrai et al., 2020). Prognosis likely worsens gradually as tumor size increases but becomes significantly poorer when regional lymph node involvement occurs (Cassali et al., 2024).

Traditionally, veterinary studies have inferred the impact of treatment on QoL from objective measures of physical side effects (Smith et al., 2019). Assessing QoL in human neonates, infants, individuals with mental disabilities, and critically ill patients poses significant challenges, as these individuals cannot directly communicate their experiences. To address this limitation, instruments were developed to obtain information from proxy informants such as parents, spouses, partners, caregivers, and healthcare providers (Schnabel et al., 2023). Similarly, in animals, information must be gathered indirectly from the person most familiar with the animal (Fan, 2014; Smith et al., 2019; Yazbek & Fantoni, 2005). This approach was applied in the present study to ensure comprehensive data collection.

Although previous studies have evaluated caregiver perceptions and the QoL of pets undergoing cancer treatment (Smith et al., 2019), to the best of current knowledge, none have specifically focused on surgical management. This study was therefore conducted in recognition of the importance of this disease in small animal surgery. While no standardized definition of QoL exists for dogs, most authors agree that it represents a continuum reflecting both physical and mental well-being.

The HHHHHMM Scale (Villalobos, 2011) demonstrated a correlation between clinical stage and QoL, as reflected by distinct preoperative scores across different stages. This difference was no longer observed after surgery, suggesting that MMNs negatively affect QoL, similar to findings in humans (Graner et al., 2010). Postoperative results indicated both improvement and normalization of QoL across stages, confirming that surgical intervention enhances well-being regardless of disease severity. The significant improvement in clinical scores among animals classified as stages II, III and IV further supports the effectiveness of surgical treatment.

The QEHQ (Yazbek & Fantoni, 2005) produced similar results. The initial assessment showed differences in QoL among dogs at different stages, which were no longer present 45 days after surgery. These findings highlight the role of surgery in improving QoL, particularly in dogs with stage III and IV disease, consistent with observations from the HHHHHMM Scale. The results also suggest that the procedure effectively reduces pain, mirroring outcomes reported in human studies (Graner et al., 2010).

Stages III and IV showed the most positive responses to surgery on both scales. This outcome is likely related to the systemic nature of the neoplasms, which affect the body in a chronic and widespread manner, influencing multiple physiological and behavioral functions (Yazbek & Fantoni, 2005). In this context, surgery can serve a palliative role, contributing to improved QoL. Comparing these findings with those from previous studies remains challenging because of methodological differences. The present study focuses primarily on clinical staging, a key parameter used to estimate survival, evaluate the relationship between disease stage and age, and guide prognostication and therapeutic decision-making (Cassali et al., 2024).

In this study, pain emerged as a major concern on both scales, particularly on the HHHHHMM Scale (Villalobos, 2011), where 46.6% of the dogs scored 5 out of 10 or lower, indicating reduced QoL. The prevalence of pain was comparable to that reported in humans, where approximately 50% of patients with MMNs experience pain (Graner et al., 2010). In human studies, pain occurs in about 30% of patients in early stages and increases to 65–85% as the disease advances (Fan, 2014). These findings confirm that pain remains a substantial and persistent burden associated with neoplasia in dogs.

Pain associated with cancer negatively impacts QoL and physiological functions such as cellular metabolism and immune activity, making its relief a therapeutic priority (Fan, 2014). The mechanisms through which neoplasms induce pain are diverse and include direct effects such as tissue damage, compression, and invasion, as well as indirect effects mediated by paraneoplastic factors (Williams & MacDonald-Dickinson, 2023). Activation of nociceptors, tissue injury, inflammation, peripheral and central sensitization, and wind-up phenomena often limit the effectiveness of superficial pain management strategies (Villalobos, 2011). In this study, however, a marked improvement in QoL was observed 45 days after surgery.

Conversely, according to the QEHQ (Yazbek & Fantoni, 2005), 33.3% of caregivers initially reported that the disease had a significant negative impact on the QoL of their dogs, reflected by lower scores. After surgery, this perception changed, with 73.3% indicating that the disease no longer interfered with the well-being of their pets. Overall, the mean QEHQ scores improved markedly from pre- to post-surgery, approaching values observed in healthy animals (Yazbek & Fantoni, 2005).

This study presents a minor limitation. Because the HHHHHMM Scale has not been officially validated in Portuguese for use in Brazil, interpretation of the final QoL score should be approached cautiously when making critical decisions, particularly those concerning life and death.

## Conclusion

Unilateral mastectomy and regional lymphadenectomy positively influence the QoL of dogs with MMNs, particularly those in stages III and IV. Initial assessments revealed compromised QoL and a high prevalence of pain, both of which improved significantly 45 days after surgery. These findings highlight the importance of early diagnosis and timely intervention, as well as the value of using proxy informants to assess QoL in dogs.

## References

[B001] Albring K. M. F., Ceolin S., Costa A. R. D. (2023). Exposição à agrotóxicos e câncer: Revisão integrativa de literatura. Research, Society and Development.

[B002] Burrai G. P., Gabrieli A., Moccia V., Zappulli V., Porcellato I., Brachelente C., Pirino S., Polinas M., Antuofermo E. (2020). A statistical analysis of risk factors and biological behavior in canine mammary tumors: A multicenter study. Animals.

[B003] Caputo L. F. G., Gitirana L. B., Manso P. P. A., Molinaro E., Caputo L., Amendoeira M. R. R. (2010). Conceitos e Métodos para Formação de Profissionais em Laboratórios de Saúde.

[B004] Cassali G. D., Nakagaki K. Y. R., Jark P. C., Horta R. D. S., Arias A. C., Tellado M. N., Estrela-Lima A., Sueiro F. A. R., Reys M. P. D., Wronski J. G., Sierra O. R., Kristancic E. A., Decuadro A., Gómez J. L. Á. (2024). Consensus on the diagnosis, prognosis, and treatment of canine and feline mammary tumors: Solid arrangement – 2023. Brazilian Journal of Veterinary Pathology.

[B005] Cassali G. D., Jark P. C., Gamba C., Damasceno K. A., Estrela-Lima A., Nardi A. B. D., Ferreira E., Horta R. S., Firmo B. F., Sueiro F. A. R., Rodrigues L. C. S., Nakagaki K. Y. R. (2020). Consensus regarding the diagnosis, prognosis and treatment of canine and feline mammary tumors - 2019. Brazilian Journal of Veterinary Pathology.

[B006] Fan T. M. (2014). Pain management in veterinary patients with cancer. The Veterinary Clinics of North America. Small Animal Practice.

[B007] Gaboardi S. C., Candiotto L. Z. P., Ramos L. M. (2019). Perfil do uso de agrotóxicos no sudoeste do Paraná (2011-2016). Revista Nera.

[B008] Gamba C. O., Ferreira E., Salgado B. S., Damasceno K. A., Bertagnolli A. C., Nakagaki K. Y. R., Cassali G. D., Cassali G.D., Ferreira E., Campos C. B. (2017). Patologia Mamária Canina do Diagnóstico ao Tratamento.

[B009] Graner K. M., Costa A. L., Rolim G. S. (2010). Dor em oncologia: Intervenções complementares e alternativas ao tratamento medicamentoso. Temas em Psicologia.

[B010] Kristiansen V. M., Peña L., Díez Córdova L., Illera J. C., Skjerve E., Breen A. M., Cofone M. A., Langeland M., Teige J., Goldschmidt M., Sørenmo K. U. (2016). Effect of ovariohysterectomy at the time of tumor removal in dogs with mammary carcinomas: A randomized controlled trial. Journal of Veterinary Internal Medicine.

[B011] MacPhail C., Fossum T. W., Fossum T. W. (2019). Small Animal Surgery.

[B012] Mencalha R., Generoso C. S., Souza D. S. (2019). Bloqueio analgésico intervencionista em cão com síndrome da cauda equina: Relato de caso. BrJP.

[B013] Nosalova N., Huniadi M., Horňáková Ľ., Valenčáková A., Horňák S., Nagoos K., Vozar J., Cizkova D. (2024). Canine mammary tumors: Classification, biomarkers, traditional and personalized therapies. International Journal of Molecular Sciences.

[B014] Nunes F. C., Campos C. B., Bertagnoli A. C., Cassali G.D., Ferreira E., Campos C. B. (2017). Patologia Mamária Canina do Diagnóstico ao Tratamento.

[B015] Santos S. B. G., Sandri G., Oliveira K. C. S., Rossi M. G., Silva D. F. S., Nunes A. T., Jesus L. V., Corona C. F., Lima F. A. A., Carvalho K. A. R., Dalmolin C., Panis C., Benvegnu D. M. (2024). Mechanisms of Immune dysregulation induced by pesticide exposure and their implications for cancer: A systematic review. Advances in Clinical Toxicology.

[B016] Schnabel A., Lordick F., Oberth P., Neuschulz M., Lehmann-Laue A., Mehnert-Theuerkauf A., Hinz A. (2023). Supportive care needs and health-related quality of life in cancer patients receiving palliative care. Frontiers in Psychology.

[B017] Silva H. D. C., Oliveira A. R., Horta R. D. S., Rassele Merísio A. C., Sena B. V., Carlos De Souza M. C., Flecher M. C. (2019). Epidemiology of canine mammary gland tumours in Espírito Santo, Brazil. Acta Scientiae Veterinariae.

[B018] Smith P. A. D., Burnside S., Helm J. R., Morris J. S. (2019). Owner perceptions of radiotherapy treatment for veterinary patients with cancer. Veterinary and Comparative Oncology.

[B019] Sorenmo K. U., Durham A. C., Kristiansen V., Pena L., Goldschmidt M. H., Stefanovski D. (2019). Developing and testing prognostic bio‐scoring systems for canine mammary gland carcinomas. Veterinary and Comparative Oncology.

[B020] Sorenmo K. U., Worley D. R., Zappulli V., Vail D. M., Thamm D. H., Liptak J. M. (2020). Withrow MacEwen’s Small Animal Clinical Oncology.

[B021] Testoni I., De Vincenzo C., Campigli M., Caregnato Manzatti A., Ronconi L., Uccheddu S. (2023). Validation of the HHHHHMM scale in the Italian context: Assessing pets’ quality of life and qualitatively exploring owners’ grief. Animals.

[B022] The Jamovi Project (2022). Jamovi (Version 2.3).

[B023] Vazquez E., Lipovka Y., Cervantes-Arias A., Garibay-Escobar A., Haby M. M., Queiroga F. L., Velazquez C. (2023). Canine mammary cancer: State of the art and future perspectives. Animals.

[B024] Villalobos A. E. (2011). Quality-of-life assessment techniques for veterinarians. The Veterinary Clinics of North America. Small Animal Practice.

[B025] Williams K., MacDonald-Dickinson V. (2023). Progress in palliation: Managing pain caused by cancer in veterinary medicine. The Canadian Veterinary Journal = La Revue Vétérinaire Canadienne.

[B026] Yazbek K. V. B., Fantoni D. T. (2005). Validity of a health-related quality-of-life scale for dogs with signs of pain secondary to cancer. Journal of the American Veterinary Medical Association.

[B027] Zappulli V., Peña L., Rasotto R., Goldschmidt M. H., Gama A., Seruggs J. L., Kiupel M., Zappulli V., Peña L., Rasotto R., Goldschmidt M.H., Gama A., Seruggs J.L., Kiupel M. (2019). Surgical Pathology of Tumors of Domestic Animals.

